# The two-step approach to allogeneic hematopoietic stem cell transplantation

**DOI:** 10.3389/fimmu.2023.1237782

**Published:** 2023-09-01

**Authors:** Sikemi Ibikunle, Dolores Grosso, Usama Gergis

**Affiliations:** Department of Medical Oncology, Kimmel Cancer Center, Thomas Jefferson University, Philadelphia, PA, United States

**Keywords:** two-step approach, haploidentical, matched related, stem cell transplantation, cyclophosphamide tolerization

## Abstract

Allogeneic hematopoietic stem cell transplantation (HSCT) provides the only potentially curative option for multiple hematological conditions. However, allogeneic HSCT outcomes rely on an optimal balance of effective immune recovery, minimal graft-versus-host disease (GVHD), and lasting control of disease. The quest to attain this balance has proven challenging over the past few decades. The two-step approach to HSCT was conceptualized and pioneered at Thomas Jefferson University in 2005 and remains the main platform for allografting at our institution. Following administration of the transplant conditioning regimen, patients receive a fixed dose of donor CD3+ cells (HSCT step one-DLI) as the lymphoid portion of the graft on day -6 with the aim of optimizing and controlling T cell dosing. Cyclophosphamide (CY) is administered after the DLI (days -3 and -2) to induce donor-recipient bidirectional tolerance. On day 0, a CD34-selected stem cell graft is given as the myeloid portion of the graft (step two). In this two-step approach, the stem cell graft is infused after CY tolerization, which avoids exposure of the stem cells to an alkylating agent, allowing rapid count recovery. Here, the two-step platform is described with a focus on key results from studies over the past two decades. Finally, this review details lessons learned and current strategies to optimize the graft-versus-tumor effect and limit transplant-related toxicities.

## 1 Introduction

In the early era of haploidentical (HI) hematopoietic stem cell transplantation (HSCT), the infusion of T cell depleted (TCD) grafts was used to successfully cross the major histocompatibility (MHC) barrier and avoid catastrophic graft versus host disease (GVHD). TCD approaches continue to be used successfully today, with strategies to reduce infectious sequela from delayed immune reconstitution, such as adoptive therapy with virus-specific T cells, employed to increase survival rates (1). However, in 2002, the bone marrow transplant group at Johns Hopkins published one of the first reports of the use of post-transplant cyclophosphamide (CY) as a T-cell attenuation strategy in non-myeloablative HI-HSCT to avoid graft rejection and significant GVHD. In this approach, CY was administered both before and after the allogeneic graft to suppress alloreactive T cells and promote regulatory T cell reconstitution in lieu of the TCD strategy (2). This approach was associated with very low rates of both chronic and acute GVHD and, in contrast to TCD approaches, robust immune reconstitution. Since this early report, post-transplant CY has expanded to include regimens of increased intensity using mobilized peripheral blood stem cell grafts to decrease relapse rates, which were associated with its original application (3).

The idea for a two-step approach in HI-HSCT using CY as GVHD prophylaxis arose from a reluctance to expose the donor stem cell product to an alkylating agent. To accomplish this, after conditioning, an unmanipulated CD3+ dose (DLI) was infused as step one of the HSCT. CY was administered 2 days later for GVHD prophylaxis. This 48-hour period between the administration of MHC incompatible cells and CY administration allowed for the development of tolerance based on the original murine studies (4) with CY and data from Hopkins (5). Twenty-four hours after the last dose of CY, a CD34 selected stem cell product was infused (step two of the HSCT) having avoided exposure to CY. Two additional benefits from this two-step process, both with the potential to reduce relapse rates, were realized. The first was the ability to manipulate the dose of tolerized T cells and the second was the avoidance G-CSF effects on the DLI product. In the former, the content of T-cells in the graft was no longer affected by the need to obtain a specific stem cell dose and could be independently titrated for potentially higher graft versus tumor (GVT) effects. In the latter, allogeneic donors initiated G-CSF for stem cell mobilization prior to DLI collection thereby avoiding Th2 effects on T cell cellular immunity. This effect was further recapitulated with the use of GM-CSF in recipients post HI-HSCT. While the design of the two-step approach occurred in 2005, the first official clinical trial did not open at Thomas Jefferson University until 2006. At that time, relapse was the primary cause of treatment failure in the transplant program and therefore a myeloablative regimen was used for conditioning. The original clinical trial was a phase I/II trial testing the safety and efficacy of the DLI dose in step 1 of the approach. The initially tested dose of 2 x 10^8^/kg CD3+ cells was found to be safe and has not been changed in the ensuing 17 years. The conclusions of this first trial were that the approach was effective especially in patients with controlled disease at the time of HI-HSCT, was associated with highly acceptable rates of GVHD, and resulted in robust immune reconstitution (6). Three additional critical pieces of information were gleaned from the trial. First, engraftment was consistent except in the presence of strong anti-donor human leukocyte antigen (HLA) antibodies, engraftment was rapid (neutrophil engraftment within 9-15 days), and unexpectedly, the 2 x 10^8^/kg DLI dose was associated with a haploimmunostorm which in the earlier stages of the trial had not been a recognized effect of higher T cell doses until the publication by Colvin and colleagues in 2009 (7). The haploimmunostorm was managed successfully with supportive care, and in later years, the addition of inferior vena cava monitoring helped with fluid balance during this time.

In the decades that followed the initial two-step clinical trial, Jefferson investigators have published additional studies detailing evolutions in the two-step methodology including the extension of the platform from haploidentical (HI) donor grafts to include matched-related donor (MRD) grafts, a revision of the understanding of the role of CY and timing of T cell administration, and the adoption of strategies to address donor-specific anti-HLA antibodies (DSA) and specific mechanisms of relapse. Key results from these studies are discussed below and their findings integrated into updated recommendations on the use of this approach based on over 400 two-step transplant procedures performed to date.

## 2 The two-step approach

In the initial phase I/II clinical trial, 27 patients were enrolled with hematological malignancies (HM) and a median age of 52 years old (6). Patients received a myeloablative conditioning (MAC) regimen of 12 Gy of total body irradiation (TBI) in eight fractions over 4 days on days -9 to -6. After the last dose of TBI, patients received an infusion of 2.0 x 10^8/kg of donor T cells (DLI; step 1).

The optimal T cell dose was determined during phase I of the clinical trial. The optimized T-cell dose was selected given its association with consistent and high rates of engraftment, robust immune reconstitution, and low rates of grade III/IV aGVHD.

During the two days following DLI (which consist of rest days on day -5, day -4), patients typically experienced high, non-infectious fever (median, 103.8 deg F), diarrhea, and less often rash – a phenomenon termed “haploimmunostorm.” This occurrence is driven by the *in vivo* activation of a large quantity of alloreactive lymphocytes.

**Figure 1:**
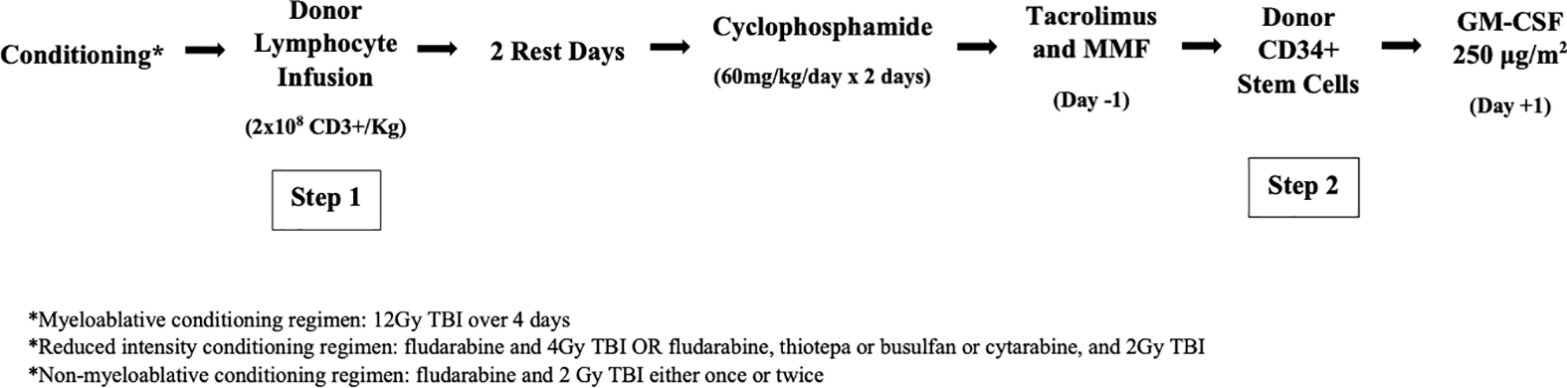
The two-step approach to allogenic HSCT. After conditioning the donor lymphocyte infusion containing 2x10^8^ CD^3^ +cells/kg is infused (step 1) followed by 2 rest days and then 2 days of high-dose cyclophosphamide. Tacrolimus and MMF are initiated on day -1.CD34+ stem cells are then infused (step 2) followed by GM-CSF on day +1.

On days -3 and -2, CY 60 mg/kg/d is given to promote bidirectional T-cell tolerization based on the findings of Mayumi et al. (8) and investigators at Johns Hopkins (9). While the post-DLI infusion fever was impervious to antipyretics and other supportive therapies, it invariably resolved with the second day of CY. The unique efficacy of CY in this instance reflects the drug’s ability to dramatically reduce the population of activated, alloreactive T cells *in vivo*. After a rest day (day -1), patients received step 2 of the transplant, donor CD34+ selected cells, on day 0. For additional GVHD prophylaxis, patients were given tacrolimus and mycophenolate mofetil (MMF). In patients without GVHD, MMF was discontinued on day +28 and tacrolimus was tapered starting on day +60. GM-CSF 250 μg/m^2^ was started on day +1. The myeloablative conditioning (MAC) regimen used in the first trial is depicted in **Figure 1**. In later trials, reduced intensity conditioning (RIC) and nonmyeloablative (NMA) conditioning regimens were used, which are also included in **Figure 1**.

## 3 The initial study of the two-step approach

In the initial phase I/II clinical trial, reliable and rapid engraftment was achieved, with a median of 12 days to achieve absolute neutrophil count (ANC) >500/uL and 20.5 days for platelets >20,000/uL (6). Study results are summarized in **Table 1**, and data for the outcomes of all of the published two-step trials are summarized in **Table 2**. Time to engraftment was shorter than other studies of HI HSCT that used post-transplant cyclophosphamide, where the median was 17-18 days for neutrophils and 22-37 days for platelets (10, 11). As a result, the period of pancytopenia patients experienced was shorter, translating to decreased risk of hospital-acquired infection, lower transfusion burden, and reduced hospital length of stay (LOS) and health utilization resources.

**Table 1:** Studies on the Jefferson two-step approach to allogeneic HSCT.

Author	Study Participants	Study design and aims	Results
**Grosso et al., 2011**	27 patients with HM or aplastic anemia undergoing HI HSCT	Prospective study Optimal T-cell dose, OS, PFS, relapse, NRM, GVHD, time to engraftment	A high, fixed dose of haploidentical T cells was associated with promising outcomes including high OS
**Grosso et al., 2015**	28 patients with HM without morphologic disease undergoing HI HSCT	Prospective study OS, PFS, relapse, NRM, GVHD, time to engraftment	The results corroborated prior evidence that patients with earlier stage disease experience a higher rate of disease-free survival
**Gaballa et al., 2016**	87 patients with HM or aplastic anemia undergoing HI and MRD HSCT	Retrospective study^1^ OS, PFS, relapse, NRM, GVHD, time to engraftment, CMV reactivation	Early immune recovery and clinical outcomes were similar in both haploidentical and matched-related SCT recipients
**Geethakumari et al., 2017**	92 patients with AML or MDS undergoing HI HSCT	Retrospective study^2^ Relapse	Maternal recipients had the highest risk of relapse followed by sibling recipients, then paternal recipients
**Grosso et al., 2017**	50 patients with AML undergoing HI HSCT	Retrospective study^3^ Prevalence of aUDP-6p in relapsed disease	aUPD-6p was found frequently in patients with late relapse of AML
**Grosso et al., 2020**	46 patients with HM undergoing MRD HSCT	Prospective study OS, PFS, relapse, NRM, GVHD, time to engraftment	Use of high doses of T cells with cyclophosphamide tolerization resulted in high OS with very low incidence of GVHD and NRM
**Yang et al., 2022**	60 patients with lymphoid malignancies undergoing HI and MRD SCT	Retrospective study^2^ OS, PFS, relapse, NRM, GVHD, time to engraftment	Compared to registry studies from EBMT and CIBMTR, PFS was higher, and OS was either higher or comparable. Neutrophil engraftment was superior and platelet recovery was rapid.
**Xia et al., 2022**	76 patients aged ‗65 years-old with HM undergoing HI HSCT	Retrospective study^3^ OS, PFS, NRM, relapse, GVHD, time to engraftment	Rapid engraftment and low rates of disease relapse were achieved in this group of elderly patients.

^1^ This retrospective study included patients in the prospective studies by Grosso et al., 2011 and Grosso et al., 2015. Additionally, it included a subset of patients in the prospective study by Grosso et al., 2020 and a subset of patients in multiple prospective trials whose data have not been published independently. All patients were enrolled in prospective trials, ensuring uniformity in the supportive care and treatment received.

^2^These retrospective studies included a subset of patients in the prospective studies by Grosso et al., 2011, Grosso et al., 2015, and Grosso et al., 2020, as well as a subset of patients in multiple prospective trials whose data have not been published independently.

^3^These retrospective studies included a subset of patients in the prospective studies by Grosso et al., 2011 and Grosso et al., 2015 as well as a subset of patients in multiple prospective trials whose data have not been published independently.

**Table 2:** Outcomes of Jefferson two-step approach to allogeneic HSCT.

	*Time to neutrophil engraftment (days)*	*Time to platelet engraftment (days)*	*WBC engraftment %*	*Platelet engraftment %*	*Grade II-IV GVHD*	*Grade III-IV GVHD*	*Chronic GVHD*	*Deaths from GVHD*	*Deaths from infection*	*Median CD3+/CD4+ count (cells/uL)*	*NRM*	*Rate of relapse*	*OS*
*Grosso et al., 2011*	12	20.5	85%^1^	74%^1^	59%	7%	15%	None	3	34 at 28 days, 105 at 100 days	22% at 2 years	32% at 2 years	54% at 1 year, 48% at 3 years
*Grosso et al., 2015*	11	17	100%	100%	39%	4%	21%	None	None	74 at 28 days, 148 at 90 days	4% at 2 years	21% at 2 years	89% at 1 year, 77% at 2 years
*Gaballa et al., 2016*	11 (HI), 11 (MR)	17 (HI), 18 (MR)	96% (HI), 100% (MR)	96% (HI), 100% (MR)	40% (HI), 8% (MR)	6% (HI), 4% (MR)	19% (HI), 12% (MR)	None	2 (HI), 0 (MR)	50 (HI) and 48 (MR) at 28 days, 134 (HI) and 166 (MR) at 100 days	10% (HI) and 4% (MR) at 3 years	22% (HI) and 27% (MR) at 3 years	70% (HI) and 71% (MR) at 3 years
*Grosso et al., 2020*	11	17	98%	98%	13%	4%	9%	None	None	61 at 28 days, 177 at 90 days	4% at 1 and 5 years	31% at 2 years	89% at 1 year, 66% at 5 years
*Yang et al., 2022*	11	16	100%	100%	45%	5%	15%	4^2^	6^2^	Not available	30% at 3 years	12% at 3 years	63% at 3 years
*Xia et al., 2022*	11	18	100%	100%	39.5%	11%	19%	4	16	Not available	43.5% at 3 years	21% at 3 years	37% at 3 years

^1^Two multiparous female patients with multiple HLA donor-specific antibodies rejected grafts from their daughters. One of these patients received 4 doses of rituximab and apheresis then a reduced intensity conditioning regimen and achieved successful engraftment using the original donor.

^2^At extended follow-up of 6 years.

MR, matched-related.

The rate of grade II-IV aGVHD was 59%, primarily due to grade II skin GVHD. There were no deaths from GVHD. Rates of grade III-IV aGVHD and cGVHD were low at 7% and 16%, respectively. There were no cases of extensive cGVHD. Rates of grade II-IV aGVHD were higher in this study than in other HI-HSCT trials using CY tolerization while rates of grade III-IV aGVHD and cGVHD were comparable or lower (10–12). The higher rates of grade II-IV aGVHD may be explained by the 5-fold higher T cell dosing and preferential selection of the most alloreactive available donor. Notably, GVHD was mainly limited to the skin and readily managed with corticosteroids and photopheresis in nearly all patients.

Rapid and durable immune reconstitution was achieved: median CD3+/CD4+ counts at 28 and 100 days post-HSCT were 34 cells/uL and 105 cells/uL.

Three patients died of infection, and three other patients died from regimen-related toxicity. Non-relapse mortality (NRM) was 22%. The most common cause of death in these patients was relapsed disease, principally in patients with AML or MDS with morphologic evidence of disease at the time of transplant. The rate of relapse was 32% at 2 years. No patients with lymphoid disease experienced relapse.

OS was 54% at 1 year and 48% at 3 years. OS in patients without evidence of morphologic disease at the time of transplant was 75% at 3 years. OS in patients with evidence of morphologic disease at the time of transplant was 27% at 3 years. All surviving patients at the 3-year posttransplant mark had no evidence of relapsed disease. OS was higher than that in similar HI-HSCT trials using CY tolerization where 1-year OS was 46-48% (11, 12).

## 4 Outcomes of the two-step approach in specific patient populations

### 4.1 Outstanding outcomes of patients with no evidence of disease at transplantation

A subset of patients with earlier stage disease in the initial two-step trial experienced excellent outcomes with a high OS of 75% at 3 years (6). These results suggested that the two-step approach to HI HSCT represents a promising strategy for patients with earlier stage disease who lack MRDs. Thus, a follow-up trial was performed to assess the reproducibility of the results.

In this trial, 28 additional patients with HM and no morphologic evidence of disease were enrolled. The median age was 47 years (13). Patients were treated with two-step HSCT as previously described.

Rapid engraftment proved reproducible with a median of 11 days for neutrophils and 17 days for platelets. The NRM was 0% at 100 days and 4% at 2 years; a single patient died of NRM causes. No patients died from infection or GVHD. Immune reconstitution was swift and durable: median CD3+/CD4+ counts at 28 and 90 days post-HSCT were 74 cells/uL and 148 cells/uL, respectively.

The rate of grade II-IV GVHD was 39%, again primarily due to skin GVHD. Rates of grade III-IV aGVHD were low at 4% (n=1), and the cumulative incidence of cGVHD was 21% at 2 years. There were no cases of extensive cGVHD and no deaths from GVHD. Skin GVHD responded well to corticosteroids and photopheresis. The low rates of grade III-IV aGVHD and extensive cGVHD were attributed to the effective CY tolerization of T lymphocytes and the use of double GVHD prophylaxis with MMF and tacrolimus.

OS and PFS at 2 years were similar to findings in this subgroup in the initial two-step trial. OS was 89% at 1 year and 77% at 2 years. PFS at 1 and 2 years was 79% and 74%, respectively. Relapsed disease was the only cause of death in the 27 of 28 patients that survived through discharge. Relapse occurred at a rate of 21% at 2 years. None of the subset of 12 patients without morphologic disease in the initial trial and only 1 patient in this trial developed relapsed malignancy more than 12 months after HSCT.

The low toxicity of the regimen and the robust immune recovery attained was evidenced by a low NRM rate; there was no mortality from rejection, infection, or GVHD, further supporting the use of two-step HI HSCT in patients in morphologic remission. Whereas 2 patients in the initial trial experienced graft rejection due to donor HLA antibodies, no patients in this trial had HLA antibodies against donor antigens, and 100% engraftment was achieved.

The eligibility criteria were updated for this follow-up clinical trial to increase patient safety: restrictions on organ function, Karnofsky Performance Status, and HCT-CI criteria were tightened to ensure patients would be able to manage the aggressive hydration and the positive fluid balance required to support patients during the haploimmunostorm period.

### 4.2 The two-step approach is well tolerated in elderly patients

Xia et al. reported the two-step experience in elderly patients – a population in which transplant and outcome data is limited (14). Historically, allogeneic (allo) HSCT was not offered to older patients due to concern for high peri-transplant mortality, high relapse, and worse OS. However, with advances in HSCT, the share of patients ‗ 65 years undergoing HSCT grew to 27% by 2020 compared to 2% in 2000 according to the Center for International Blood and Bone Marrow Transplant Research (CIBMTR) (15). Outcomes have been promising though a larger body of evidence is needed.

Seventy-six consecutive patients ‗ 65 years of age with HM underwent HI HSCT using the two-step approach between 2007 and 2021. Median age was 69 years (range 65-78 years). Patients received either myeloablative conditioning (12gy TBI) or reduced intensity conditioning (fludarabine and 4 gy TBI or fludarabine, either thiotepa or busulfan, and 2gy TBI). The majority of patients received RIC (n=65) while the remainder (n=11) received MAC.

All evaluable patients achieved successful engraftment. Median time to engraftment was short, with neutrophil engraftment at 11 days and platelet engraftment at 18 days. Two patients, both with MDS, experienced secondary graft failure.

OS and PFS were promising at 37% and 36% at 3 years, respectively. Survival outcomes were similar to those in previous studies with comparable age groups that used MRDs (16–20). However, the NRM for the cohort transplanted between 2015 and 2021 (29% and 37% at 1 year and 3 years, respectively) were akin to or better than the NRM in previous studies (33%-45% at 2 years). This improvement in NRM in later cohorts can be mainly attributed to improved supportive care (e.g., posaconazole and letermovir).

Notably, higher disease risk index score and HSCT comorbidity index were associated with lower OS; age, however, was not predictive of survival.

At 3 years, the NRM was high at 43.5%, and relapse rate was low at 21%. Of note, no patient relapsed after year two. The primary cause of death was infection (16 of 47 deaths) followed by relapsed disease (14 of 47 deaths) followed by treatment-related toxicity (10 of 47 deaths).

Grade II-IV aGVHD occurred at a rate of 39.5% at 6 months and at 1 year, and grade III-IV aGVHD occurred at a rate of 11% at 6 months and at 1 year. While the majority (78%) of aGVHD involved only the skin, 10 patients developed GI aGVHD, and 3 patients developed liver aGVHD. Four patients died from complications of aGVHD involving the skin and either the gut or liver. cGVHD was 16% at 1 year and 19% at 2 years. The incidence of severe cGVHD was 6% with no attributable deaths.

These findings indicated that select elderly patients have the potential to attain disease control and extended survival using the two-step approach. Ongoing refinements in this protocol and candidate selection are expected to continuously improve transplant outcomes.

### 4.3 Excellent outcomes in patients with lymphoid malignancies

Despite recent major advances in lymphoma therapy including CAR-T cells and pathway inhibitors, allo HSCT remains the only potentially curative option for patients with relapsed/refractory lymphoid malignancies.

Sixty patients with high-risk lymphoid malignancies who underwent two-step allo HSCT from 2008 to 2020 were analyzed retrospectively (21). Underlying malignancies included diffuse large B cell lymphoma (28%), chronic lymphoblastic leukemia (17%), follicular lymphoma (13%), and Hodgkin lymphoma (12%). The median age was 56 years. Conditioning regimens included MAC (35%), RIC (58%), and NMA (7%) conditioning. As this study took place after the two-step platform was extended to include MRD stem cell grafts, 18% of patients received a MRD graft. Most patients had evidence of disease at the time of transplant (60%).

All patients achieved successful engraftment. Neutrophil and platelet engraftment occurred rapidly at a median of 11 days and 16 days, respectively. In other lymphoma allo HSCT studies, median time to neutrophil engraftment ranged from 13 to 21 days (22–24), including one study using post-transplant cyclophosphamide where the median time was 21 days (25).

OS and PFS were 63% and 60% at 3 years, respectively. The relapse rate was 12% at 3 years, and the NRM was 30% at 3 years. NRM was the primary cause of death and included death from GHVD, infection, organ toxicity, and non-transplant-related causes. Compared to a contemporaneous CIBMT registry study of patients with lymphoma undergoing HSCT with post-transplant CY, the 3-year DFS in this study was superior (60% versus 48% in the registry study), and the 3-year OS was comparable (63% versus 61% in the registry study) (26). The relapse rate was low at 12% at 3 years compared to 37% in the registry study despite 60% of patients in this trial having evidence of disease at the time of transplant.

The incidence of grade II-IV aGVHD at 1 year was 45%, and that of grade III-IV at 1 year was low at 5%. The majority of aGVHD was limited to the skin (64%). The incidence of cGVHD at three years was 15%. At 6-year follow-up, three patients had died from aGVHD with skin and gut involvement, and of the nine patients who developed cGVHD, 2 developed severe cGVHD and one died from GVHD with lung and skin involvement.

Of note, a subgroup analysis of the 17 patients with DLBCL revealed higher NRM (41%) and rates of relapse (29%) and thus lower OS (35%) at 3 years than patients with other lymphoid malignancies. Eight of these patients (47%) had disease of intermediate-risk, and eight (47%) had disease of high or very high-risk. The lower OS in these patients is reflective of the higher prevalence of heavily pretreated and chemoresistant disease in this population. This is comparable to larger registry studies despite active disease in nearly all patients in the present study (27, 28).

## 5 Outcomes of the two-step approach in matched-related SCT recipients

### 5.1 Comparing haploidentical and matched-related donor two-step HSCT

In 2008, a study was launched using the two-step approach to compare outcomes in HI HSCT and MRD HSCT using a uniquely homogeneous format: an otherwise identical transplant platform for both graft sources (29).

Typically, the heterogeneity of HSCT strategies hinders a rigorous comparison of outcomes among HI approaches and between HI HSCT vs. HSCT using other donor sources. However, the two-step strategy confers uniformity in T cell dosing, conditioning regimen, and GVHD prophylaxis strategy. Jefferson investigators hypothesized that using this method to compare outcomes in HI HSCT and MRD HSCT would lead to similar immune recovery and clinical outcomes across the two groups.

Fifty patients undergoing two-step HI HSCT were retrospectively compared to 27 patients undergoing two-step MRD HSCT for HM and aplastic anemia. Both groups received MAC, and the median age was 49 years in each group.

Early immune recovery was similar in all measured T cell subsets except for median CD3/CD8 cell count, which was higher in the MRD group at day 28 compared with the HI group (median 98 versus 39 cells). This disparity may be attributed the propensity for more rapid immune reconstitution in HLA matched HSCT due to reduced donor-host alloreactivity in this context or the negative effect of CMV reactivation on CD8+ T cells. CMV reactivation was significantly greater in the HI group as compared to the MRD group. Despite this disparity in CD3/CD8 cell count between the HI and MRD group at day 28, there were no differences in death due to infection.

Engraftment was achieved in 96% and 100% of HI HSCT and MRD HSCT patients, respectively. Engraftment was rapid at median of 11 days to ANC recovery in both groups and 17 and 18 days for median platelet recovery in HI and MRD groups, respectively. Notably, haploimmunostorm was not observed in the MRD group but occurred in nearly all patients in the HI group.

There was no significant difference in OS at 3 years, which was 70% in the HI group and 71% in the MRD group. PFS was also similar at 68% in the HI group and 70% in the MRD group. This was due to similar NRM (which was overall low at 10% in the HI group and 4% in the MRD group at 3 years) and comparable relapse rates of 21% in the HI group and 27% in the MRD group. Relapsed disease was the primary cause of death in both groups.

The low NRM differs drastically from previous outcomes with T cell-depleted HI HSCT where the NRM drew down the OS, mainly due to late immune reconstitution and high mortality from infections.

Grade II-IV aGVHD occurred significantly more frequently in the HI group than in the MRD group at the 100-day mark: the incidence was 40% versus 8%, respectively. The rates of grade II-IV aGVHD were extremely low compared to the historical range of 30-40% in MRD SCT according to a contemporaneous analysis by the CIBMTR (30, 31).

The rates of grade III-IV aGVHD were similar (6% in the HI group versus 4% in the MRD group). The rates of cGVHD at 2 years were also similar (19% in the HI group versus 12% in the MRD group). There were no deaths from GVHD in either group despite the higher incidence of GVHD in the HI group, likely because the majority of aGVHD in that group was readily controlled grade II skin GVHD.

Notably, CMV reactivation occurred more frequently in the HI group at 68% versus 19%. However, no CMV tissue disease or death from CMV infection occurred.

Though some significant differences were found in immune reconstitution and GVHD incidence, clinical outcomes were overall similar in the two groups. The two-step method furnished the opportunity to provide identical conditioning regimens, graft T cell dose, and GVHD prophylaxis strategy to facilitate this comparison. The results suggest that HI HSCT is a viable alternative to MRD HSCT.

### 5.2 Matched-related donor two-step HSCT

The use of CY to establish bidirectional tolerization of recipient and donor T cells has been associated with reduced rates of GVHD and NRM after HLA-matched HSCT; however, CY use has been linked with high incidences of relapse, with an estimated incidence of 28-44% at 2 years in HLA-matched HSCT trials (32–34). CY helps to induce bidirectional tolerance to minor histocompatibility antigen differences between HLA-matched donor and recipient T cells. Differences in minor histocompatibility antigens provide the benefit of the GVT effect but also underly the development of GVHD.

Given promising early results in HLA-partially matched recipients using CY tolerization, Jefferson investigators prospectively analyzed 46 patients with HM undergoing HLA-matched related HSCT from 2008 to 2018 using a MAC regimen combined with a high fixed dose of T-cells via the two-step approach (35). The aim was to produce a reduction in relapse rates – conferred in part by the GVT effect – while maintaining the reduction in GVHD conferred by CY tolerization.

The incidences of grade II-IV aGVHD, cGVHD, and NRM were very low – equal to or lower than those in other HLA-matched CY tolerization studies – despite using a radiation-based MAC regimen (33, 34, 36–38). Grade II-IV aGVHD was 13% at 1 year and 5 years. cGVHD occurred in 9% of patients at 1 year and 5 years. The finding of lower rates of GVHD may be attributed to the use of two GVHD prophylaxis agents (tacrolimus and MMF) in addition to CY.

The NRM was 4% at 1 and 5 years. There were no deaths from GVHD or infection. Two patients died from non-relapse-related causes including diffuse alveolar hemorrhage and pulmonary toxicity. This low incidence of NRM was responsible for the high OS (89% at 1 year and 66% at 5 years) observed in this study. Relapse was the main cause of mortality with a relapse rate of 24% at 1 year, 31% at 2 years, and 46% at 5 years despite the use of MAC and a relatively high donor T cell dose. This relapse incidence was comparable to other studies using HLA-matched donors and post-transplantation CY (37, 39).

The propensity for relapse in the MRD recipients was thought to be due to the use of 3 agents for GVHD prophylaxis: CY, tacrolimus, and MMF which when used in a setting of fewer alloreactive T cells as compared to HI-HSCT, shifted the immunological balance towards relapse.

To investigate the possibility that excessive immunosuppression may have played a role in relapse rates, a *post hoc* analysis was performed. Of note, while the lymphoid graft contains a fixed dose of T cells of 2x10^8/kg T cells (step 1), the CD34-selected portion of the graft (step 2) does contain a small residual amount of non-CY-exposed T cells at a median dose of 2.98 x10^3/kg. Unlike the DLI dose, the T cell doses contained within the myeloid portion differed among patients. The *post hoc* analysis compared patients who received less than to those who received greater than the group median of non-CY-exposed residual T cells in the CD34 product. Relapse rates were significantly lower in patients who received a greater number of non-CY-exposed T cells (19% versus 58%), and there was no concurrent increase in NRM or GVHD. Additionally, higher residual T cell content was associated with achievement of ‗98% T cell chimerism at day +28. These results do suggest a CY-induced propensity towards tolerance: one potential remedy may be to increase the number of non-CY-exposed T cells in the step 2 CD34-selected product. Jefferson investigators subsequently launched a second-generation trial based on this study to determine an appropriate and consistent T cell dose to be “added back” to the step 2 product; this study is currently ongoing.

## 6 Donor-specific anti-HLA antibody screening and management

DSA may pose a greater threat to stem cell engraftment than host T cells that survive the conditioning regimen (40). DSA are preexisting antibodies against a mismatched donor’s class I and/or class II antigens. These antibodies are associated with failure of donor stem cells to engraft (termed primary graft failure) in alternative mismatched graft sources including HI, matched/mismatched unrelated, and umbilical cord blood grafts. To mitigate the influence of DSA on transplant outcomes, the primary strategy at Jefferson is to screen for DSA and to avoid donor stem cell grafts to which the patient is alloimmunized (41). When this is not possible, DSA levels are monitored weekly, and DSA desensitization strategies are initiated at DSA >2000 MFI. The DSA sensitization strategy is as follows: patients receive a course of bortezimib/IVIG twice weekly for 2 weeks and then undergo therapeutic plasma exchange twice weekly until DSA levels decline to <2000 MFI. If DSA remains >2000 MFI, therapeutic plasma exchange is continued twice weekly through conditioning and after stem cell infusion until either DSA levels are <2000 MFI or stem cells successfully engraft. This approach has been effective in reducing high levels of DSA against multiple HLA antigens in HI, matched unrelated donor, and umbilical cord blood HSCT recipients and thus attaining successful engraftment in highly sensitized patients.

## 7 Evaluating mechanisms of relapse after HI HSCT using the two-step approach

Jefferson investigators evaluated two mechanisms of relapse known to occur in patients after HI HSCT in order to guide treatment decisions in two-step HI HSCT

### 7.1 Acquired uniparental disomy in chromosome 6p as a feature of relapse

Acquired uniparental disomy (aUPD) is a tumor escape mechanism that is known to occur in leukemic cells of patients with AML after HI HSCT (42, 43). Grosso and colleagues evaluated the prevalence of aUPD in the MHC region of chromosome 6p (aUPD-6p) in leukemic cells of adult patients with relapsed AML after two-step HI HSCT (44). A subset of patients with relapsed AML post-HI HSCT were previously reported by Vago et al. to harbor this mutation which involved a mitotic recombination event resulting in the loss of the unshared HLA haplotype and a gain of the shared HLA haplotype on the leukemic cells (45). This copy neutral loss of heterozygosity resulted in the inability of allogeneic donor cells to detect the mutated leukemic cells as these cells no longer expressed the mismatched, recognizable HLA antigens resulting in leukemic escape and disease progression. Because this type of clonal evolution renders leukemic cells undetectable to the donor, post HSCT DLI is eliminated as a treatment option for relapse. Thus, the presence of aUPD-6p in patients with relapsed leukemia is evaluated in the transplant program prior to the implementation of salvage therapy.

Fifty patients with AML undergoing two-step HI HSCT using either MAC or RIC regimens were analyzed, and 13 patients (26%) relapsed (44). One of the 13 patients was not evaluable for aUDP-6p due to low blast count at relapse. Among the evaluable relapsed patients, 6/12 (50%) exhibited aUPD-6p. The median time to relapse was longer for patients with aUPD-6p (425 days) compared to those without the mutation (180 days). Leukemic marrow and blood were tested for aUPD-6p using whole-genome single nucleotide polymorphism (SNP) arrays and HLA typing except in three patients, for whom only SNP analysis (n=2) or HLA typing (n=1) was available. aUPD-6p was considered likely if the recipient’s unshared haplotype was not detected despite the presence of recipient leukemic blasts. Active disease at the time of HSCT was identified as a risk factor for the development of aUPD-6p, similar to findings of other investigators (46). Most patients who developed aUPD-6p had high-risk or resistant AML at the time of HSCT. The prevalence of aUPD-6p was greater in this study compared to others, likely due to the high dose of T cells which has been linked to aUDP-6p in one report (46), the rapid tapering of GVHD prophylaxis leading to early and robust immune reconstitution, and the aggressiveness of this approach which may allow for the development of late mechanisms of relapse, such as aUPD-6p.

Notably, aggressive salvage therapy including AML re-induction regimens produced favorable results in the two most successfully treated patients in this series, both of whom had aUPD-6p-associated relapse. Additionally, interleukin-2 therapy and a second HSCT with an alternate donor targeting the unmutated haplotype were used as effective treatment strategies in these patients.

Overall, aUPD-6p was found frequently in patients with late relapse of AML after a two-step HI HSCT. The presence of clonal evolution of leukemic cells, including aUPD-6p, at the time of relapse provides insights into the post-HSCT immunological environment and indicates potential directions for control of relapsed disease.

### 7.2 Microchimerism as a mechanism of relapse in offspring-to-maternal HI HSCT

Geethakumari et al. evaluated the outcomes of offspring-to-maternal HI HSCT to explore the effects of microchimerism on transplant outcomes using the two-step platform (47). Microchimerism develops due to bidirectional trafficking of cells during pregnancy, leading to the presence of maternal cells in the fetus and fetal cells in the mother (48). Tolerance to non-inherited maternal antigens (NIMA) persists into adulthood in offspring. Geethakumari et al. compared HI HSCT outcomes in offspring-to-maternal, offspring-to-paternal, and sibling-to-sibling transplants. Ninety-two patients with AML or MDS who underwent HI HSCT using the two-step method and either MAC or RIC regimens were analyzed. The sample was composed of 40% paternal recipients, 37% sibling recipients, and 23% maternal recipients. The three groups exhibited similar characteristics except for a lower median donor age and higher median recipient age in the maternal and paternal groups compared to the sibling group. The rate of relapse was significantly higher in the maternal recipients than in the paternal recipients (P = 0.0001) and was higher in the maternal group than the sibling group, though the difference fell just below statistical significance (P = 0.06). Maternal relapse occurred earlier, with an incidence of 54% within the first year. In contrast, relapse incidence at 1 year was 8.5% in fathers and 26.5% in siblings. Recipient age had no significant impact on relapse incidence.

There was no significant difference in aGVHD or cGVHD risk between the recipient groups. Overall survival was lowest among maternal recipients (P = 0.029). The results suggest that NIMA tolerance may influence relapse after offspring-to-maternal HI HSCT. The present findings are currently being used to help inform donor selection at Jefferson, but additional research is needed to confirm this hypothesis in a larger sample size.

## 8 Discussion

With over 400 HI HSCT two-step procedures performed at Jefferson to date, the approach has provided a consistent and reliable backbone for HSCT in the Jefferson Cellular Therapy Program and has expanded to include a variety of regimen intensities and donor sources while keeping the fundamental part of the approach consistent.

The two-step method principally spares the CD34-selected stem cell graft from exposure to post-transplant cyclophosphamide while benefiting from the use of this agent for GVHD prophylaxis, in conjunction with tacrolimus and MMF. The approach also uniquely affords the ability to use a high, fixed dose of T cells and to limit G-CSF exposure of the graft until after donor DLI collection. Given the rationale for the two-step strategy, comparisons drawn between its results and those of other HSCT trials using post-transplant cyclophosphamide centered on time to neutrophil and platelet engraftment, and cumulative incidence of GVHD. Summarily, time to neutrophil and platelet engraftment was accelerated, by approximately one week in all studies. Notably, rates of grade II-IV aGVHD were higher than in comparative studies. However, the incidence of grade III-IV aGVHD and cGVHD was not increased, and grade II-IV GVHD was primarily limited to skin and promptly managed with corticosteroids and photopheresis in the vast majority of patients. The higher rates of grade II-IV aGVHD were attributed most likely to the 5-fold higher T cell dosing and preferential selection of the most alloreactive available donor.

Furthermore, the occurrence of graft failure was notably reduced at 3.1% across all patients undergoing the two-step procedure, in contrast to a higher incidence of 13% observed in a comparable large posttransplant cyclophosphamide study (12). The elevated incidence in the comparison study may be attributed to the utilization of bone marrow (containing fewer T cells) and a non-myeloablative approach. Though the higher T cell dose in the two-step approach is associated with an increased incidence of grade II skin GVHD, it confers a valuable trade-off of decreased risk of graft failure.

Rapid immune reconstitution and a corresponding low rate of infection in matched and mismatched grafts has been demonstrated across all conditioning intensities. These immune-related outcomes along with other outcomes were demonstrated to be similar in HI and MRD recipients when the two-step platform was used to design uniquely parallel study arms to compare both graft sources (29). This study also supported the early hypothesis of the two-step investigations that use of a fixed and optimized dose of CD3+ cells improves the ability to analyze and improve transplant outcomes.

Additionally, the incidence of graft rejection with the approach is a rare occurrence in the absence of anti-donor antibodies. At the onset of the implementation of the two-step approach, a strong association was noted between graft rejection and DSA in the Grosso et al., 2011 study. The initiation and refinement of Jefferson’s DSA mitigation protocol is expected to continue to identify highly alloimmunized patients and achieve universal, successful engraftment (41).

In the experience of Jefferson investigators and that of others, greater intensity of the haploimmunostorm is associated with worse outcomes (49, 50). An analysis of the effect of haploimmunostorm on short and long-term HI-HSCT two-step outcomes is ongoing at Jefferson. This analysis has already resulted in strategies to reduce NRM such as reducing the DLI dose from 2 x 10^8^/kg CD3+ cells to 1.5 x 10^8^/kg CD3+ cells, and the introduction of cytokine inhibitors, which had not been previously used. Across all two-step analyses, the rates of grades III-IV aGVHD and extensive cGVHD have been highly acceptable with the use of CY tolerization, despite the relatively high dose of allogenic T cells used in the regimen as compared to other HI-HSCT approaches. Reducing the intensity of the haploimmunostorm has the potential to reduce rates of GVHD further.

Similar to other transplant approaches, patients undergoing a two-step HSCT with evidence of disease have a high incidence of relapse. Reduction in relapse was addressed early in the program’s development with the recognition that HI-HSCT provided a rapid route to transplant in patients without matched related donors. Thus, patients were transplanted safely prior to the development of relapsed or resistant disease (13). Other strategies to reduce relapse in patients with high-risk disease are clearly needed. For fit patients with AML or MDS, a sequential regimen of Vyxeos (or another bridging regimen) followed by a RIC HSCT has been instituted (51) in the program. Analyses of the effect of post-two-step HSCT maintenance therapy on immune reconstitution and relapse are ongoing in high-risk patients. To address the high relapse rate in MRD HSCT, investigation adding back T cells to the CD34 graft (step 2) is underway; another approach would be to omit calcineurin inhibitors from the GVHD prophylaxis. Notably, relapsed disease was the primary cause of death in every investigation except for the Xia et al. study of patients aged ‗65 years (14), who instead experienced greater risk of infection and treatment-related toxicity than participants in the other studies, and the Yang et al. study of patients with lymphoid malignancies (21), who experienced a low rate of relapse. High rates of disease relapse were observed in patients with resistant leukemia at time of transplant in the Grosso et al., 2011 study despite a MAC regimen, rapid immune reconstitution, and use of highly HLA-mismatched donors (8). Similarly, the subset of DLBCL patients studied by Yang et al., whom had a higher prevalence of chemoresistant disease, experienced higher rates of relapse and NRM thus lower OS (21). Taken together with the excellent OS observed in patients with early-stage disease in the Grosso et al., 2015 study, these inferior outcomes in chemoresistant disease highlight the continuing need for novel variations of this approach in these subsets of high-risk patients.

Haploidentical donor selection has been informed by the analysis of Geethakumari et al. (47), and the avoidance of maternal donors is part of a consistent approach to donor selection. Finally, testing for aUPD-6p in the setting of relapse assists in the planning of post HSCT relapse in patients with acute leukemia.

In summary, the two-step approach offers a fixed dose of T cells and spares the stem cells from the effect of high dose CY. The main drawbacks are the complexity and cost of the graft acquisition as donors need to be collected twice, four days apart and a CD34 selection is required for the step 2 product. The benefits of the two-step approach include faster engraftment, shorter LOS, robust immune reconstitution, and a low incidence of grade III/IV aGVHD, cGVHD, and no extensive cGVHD. Because the regimen has 3 basic components, conditioning, DLI, and stem cell infusion, it lends itself to the ability to optimize different parts of the regimen independently of the other components. Efforts in the Jefferson Cellular Therapy Program to continue to improve the regimen as well as other components of the transplant process such as donor selection and post HSCT relapse prophylaxis continue.

## Author contributions

UG devised the project and the main concepts. SI and LG wrote the manuscript with the input of UG. All authors contributed to the article and approved the submitted version.
